# Zoledronic Acid in Osteoporotic Vertebral Compression Fractures Treated With Percutaneous Kyphoplasty: A Meta-Analysis

**DOI:** 10.3389/fsurg.2021.668551

**Published:** 2021-05-21

**Authors:** Peng Tian, Yue Liu, Zhi-jun Li, Gui-jun Xu, Xin-long Ma

**Affiliations:** ^1^Department of Traumatic Orthopedics, Tianjin Hospital, Tianjin, China; ^2^Department of Spine Surgery, Tianjin Hospital, Tianjin, China; ^3^Department of Orthopedics, Tianjin Medical University General Hospital, Tianjin, China; ^4^Department of Orthopaedics, Tianjin Hospital, Tianjin, China

**Keywords:** zoledronic acid, osteoporosis, vertebral compression fractures, percutaneous kyphoplasty, meta-analysis

## Abstract

**Background:** We performed a meta-analysis to evaluate the efficacy and safety of zoledronic acid combined with percutaneous kyphoplasty (PKP) in treating osteoporotic vertebral compression fractures (OVCFs).

**Methods:** Eligible scientific articles published prior to July 2020 were retrieved from the PubMed, Springer, ScienceDirect, and Cochrane Library databases. The statistical analysis was performed with RevMan 5.1.

**Results:** Three randomized controlled trials (RCTs) and 2 non-RCTs met the inclusion criteria. The present meta-analysis revealed that zoledronic acid combined with PKP is associated with a higher BMD, a better quality of life, less severe low back pain, and fewer additional vertebral body fractures than is percutaneous vertebral augmentation alone.

**Conclusions:** Compared with PKP only, zoledronic acid combined with percutaneous vertebral augmentation is beneficial for OVCFs.

## Introduction

Osteoporotic vertebral compression fractures (OVCFs) are a major complication of osteoporosis and can result in severe back pain, spinal deformity, the loss of mobility, a decreased quality of life and even death (1, 2). Percutaneous kyphoplasty (PKP), introduced by Garfin et al., has been clinically justified by numerous studies as an effective treatment to quickly relieve pain, correct spinal kyphotic deformities and maintain spinal stabilization in patients with acute OVCFs with persistent and severe pain (3).

Bisphosphonates (BPs) have been confirmed to prevent bone loss and increase bone quality and density by inhibiting osteoclast-mediated bone resorption and decreasing bone turnover (4). To date, the administration of bisphosphonates to treat osteoporosis has been well-studied (5, 6). In theory, the application of anti-osteoporosis agents may improve outcomes in patients treated with PKP. In a retrospective study, Seo et al. (7) found that the administration of BPs reduced the occurrence of new fractures in patients with OVCFs treated with percutaneous vertebroplasty. Recently, several clinical studies have reported the treatment outcomes of zoledronic acid combined with PKP for OVCFs. However, whether zoledronic acid is safe and efficient when combined with PKP in treating OVCFs remains controversial. Therefore, this meta-analysis was conducted to evaluate the efficacy and safety of zoledronic acid combined with PKP in treating OVCFs by analyzing the current randomized controlled trials (RCTs) and non-RCTs.

## Methods

### Search Strategy

This meta-analysis was conducted in accordance with the relevant reported items for systematic reviews and meta-analyses (PRISMA) guidelines. Potentially relevant published academic articles published from the inception of the electronic databases searched to July 2020 were retrieved from the PubMed, Springer, ScienceDirect, and Cochrane Library databases. The references of the identified articles were also searched for relevant articles. None of the studies were excluded due to language restrictions. The key words used for the search were “osteoporosis,” “vertebral compression fractures,” “kyphoplasty,” and “zoledronic acid”.

### Inclusion Criteria

Studies were considered eligible for inclusion if they met the following criteria: (1) the patients underwent PKP with OVCFs; (2) the intervention was the use of zoledronic acid, control group did not use zoledronic acid; (3) the outcomes included pain assessment, function assessment, BMD, bone metabolism parameters and complications; and (4) the study was a published RCT or non-RCT trial.

### Exclusion Criteria

We excluded studies as follows: (1) those without a control group; (2) studies with no available full-text version; (3) studies with no available outcome data; and (4) studies of revision PKP.

### Selection Criteria

Two reviewers independently screened the titles and abstracts according to the eligibility criteria. The full text of the studies that potentially met the inclusion criteria were subsequently read, and the literature was reviewed to determine suitability of final inclusion. Disagreement was resolved by consulting with a third reviewer.

### Quality Assessment

The methodological quality of the RCTs was evaluated using a modification of the generic evaluation tool described in the Cochrane handbook for systematic reviews of interventions (8). The methodological quality of non-RCTs was assessed by the methodological index for non-randomized studies (MINORS) (9).

### Data Extraction

The data were extracted from the included articles by two independent reviewers. When there was incomplete data, the corresponding author of study was contacted for details. The following information was extracted: the first author's name, publication year, intervening measures, follow-up term, pain assessment, function assessment, BMD, bone metabolism parameters, and complications. Other relevant parameters were also extracted from the individual studies. If trials reported incomplete data, authors were contacted for further information.

### Data Analysis and Statistical Methods

RevMan 5.1 (The Cochrane Collaboration, Oxford, United Kingdom) was used to analyze the pooled data. The *P*-values and *I*^2^-values from the standard chi-square test were used to estimate the level of heterogeneity. When *I*^2^ > 50%, *P* < 0.1 was considered to indicate significant heterogeneity, and a random-effects model was used for the data analysis. When *I*^2^ < 50%, *P* > 0.1 was considered to indicate non-significant heterogeneity. A fixed-effects model was used for the data analysis when non-significant heterogeneity was found. Subgroup analysis was performed when significant heterogeneity was found to investigate the sources of heterogeneity. The mean differences (MDs) and 95% confidence intervals (CIs) were determined for continuous variables. The dichotomous data are expressed as the risk differences (RDs) and 95% CIs.

## Results

### Search Results

**Figure 1:**
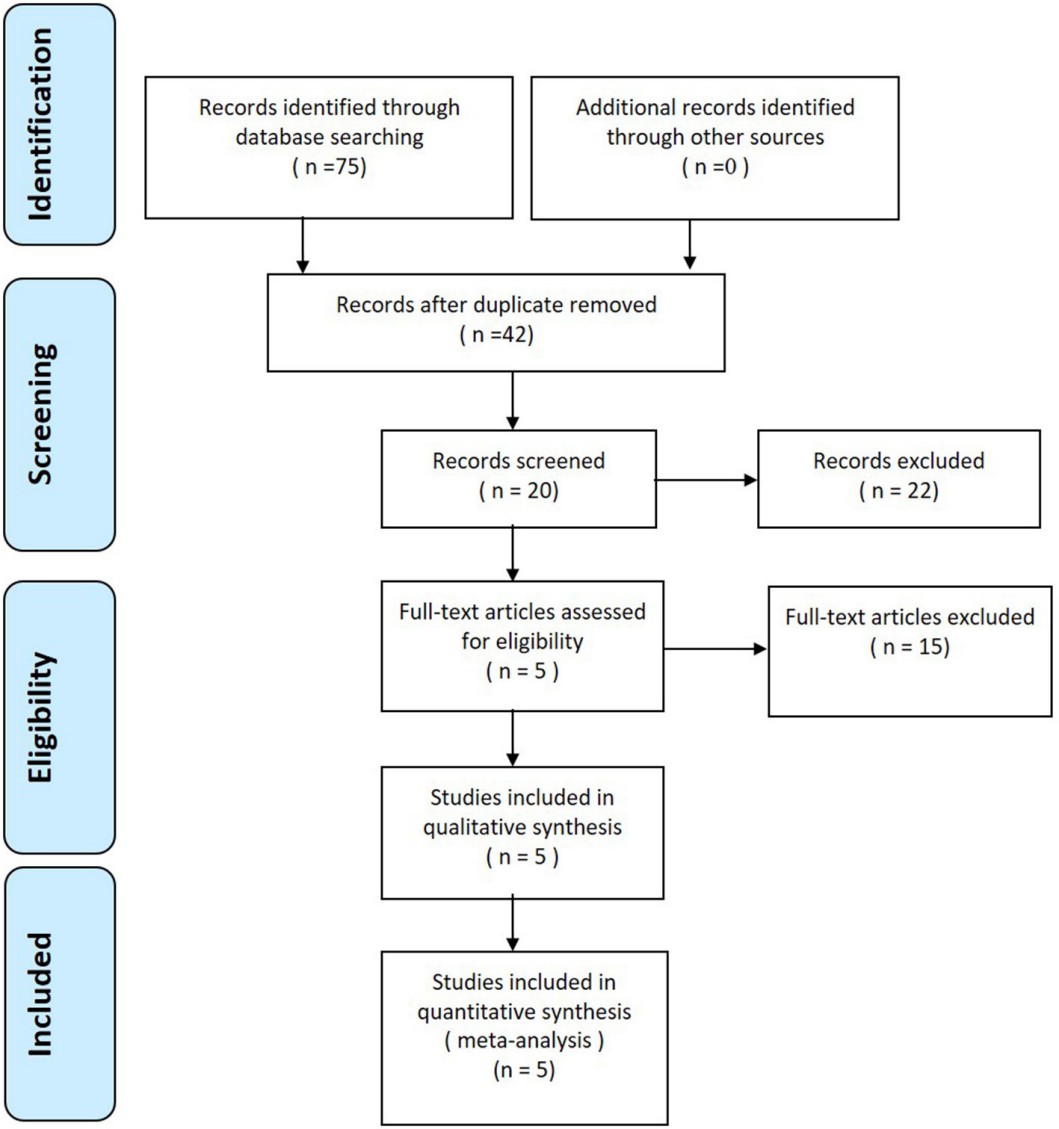
Flowchart of the study selection process.

A total of 75 studies were identified as potentially relevant. After a thorough screening of the titles and abstracts, 70 reports were excluded according to the eligibility criteria. No additional studies were identified after the references were reviewed. Finally, three RCTs (10–12) and 2 non-RCTs (13, 14) met the inclusion criteria for data extraction and meta-analysis. The search process is shown in Figure 1.

### Risk of Bias Assessment

**Figure 2:**
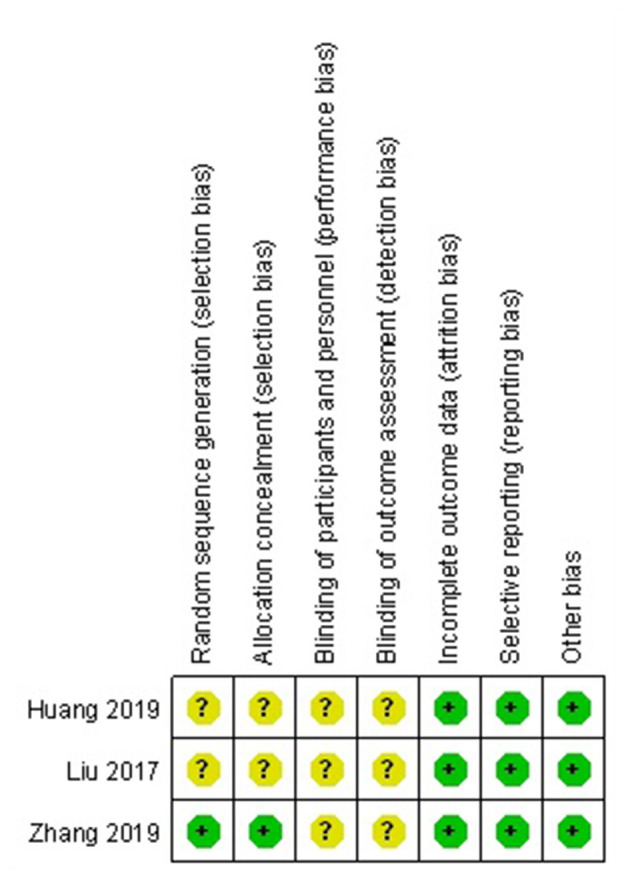
Risk of bias summary of the randomized controlled trials.

The methodological quality of RCTs was assessed according to the Cochrane handbook for systematic reviews of interventions (Figure 2). The inclusion and exclusion criteria were clearly stated in all RCTs. One RCT that was included reported adequate methodology for randomization, concealment of allocation and intent-to-treatment analysis. In addition, blinding was not described in all of the included RCTs. Unclear bias was not reported due to incomplete outcome data or selective outcomes. The MINORS score of the non-RCTs was 20. The methodological quality assessment of the non-RCTs is presented in Table 1.

**Table 1:** Quality assessment for non-randomized trials.

**Quality assessment for non-randomized trials**	**Lin et al. (14)**	**Shi et al. (13)**
A clearly stated aim	2	2
Inclusion of consecutive patients	2	2
Prospective data collection	0	0
Endpoints appropriate to the aim of the study	2	2
Unbiased assessment of the study endpoint	2	2
A follow-up period appropriate to the aims of study	2	2
<5% loss to follow-up	2	2
Prospective calculation of the sample size	0	0
An adequate control group	2	2
Contemporary groups	2	2
Baseline equivalence of groups	2	2
Adequate statistical analyses	2	2
Total score	20	20

### Study Characteristics

Demographic characteristics and other details of the included studies are presented in Table 2. In each study, the baseline characteristics of the two groups were similar. Zoledronic acid was used in all included studies.

**Table 2:** Characteristics of included studies.

**Study**	**Design**	**Intervention**	**Cases**	**Mean age**	**Female**	**Dosage**	**Follow-up**
Huang et al. (10)	RCT	ZOL + PKP	30	76.11	20	5 mg	19.46 months
		PKP	30	74.36	23	3 days after PKP	19.46 months
Lin et al. (14)	RCS	ZOL + PKP	51	75.61	49	5 mg	1.97 years
		PKP	1595	76.69	1141		1.14 years
Liu et al. (11)	RCT	ZOL + PKP	52	67.7	39	5 mg	12 months
		PKP	52	70.9	34	2 days after PKP	12 months
Shi et al. (13)	RCS	ZOL + PKP	29	77.72	13	5 mg	2 years
		PKP	34	76.65	16	3 days after PKP	2 years
Zhang et al. (12)	RCT	ZOL + PKP	50	64.6	NS	5 mg	12 months
		PKP	51	63.98	NS	2 days before PKP	12 months

*RCS, Retrospective controlled trial; RCT, Randomized controlled trial; ZOL, zoledronic acid; PKP, percutaneous kyphoplasty; F, Female; NS, Not state*.

### Outcomes of the Meta-Analysis

#### Additional Vertebral Body Fractures

**Figure 3:**
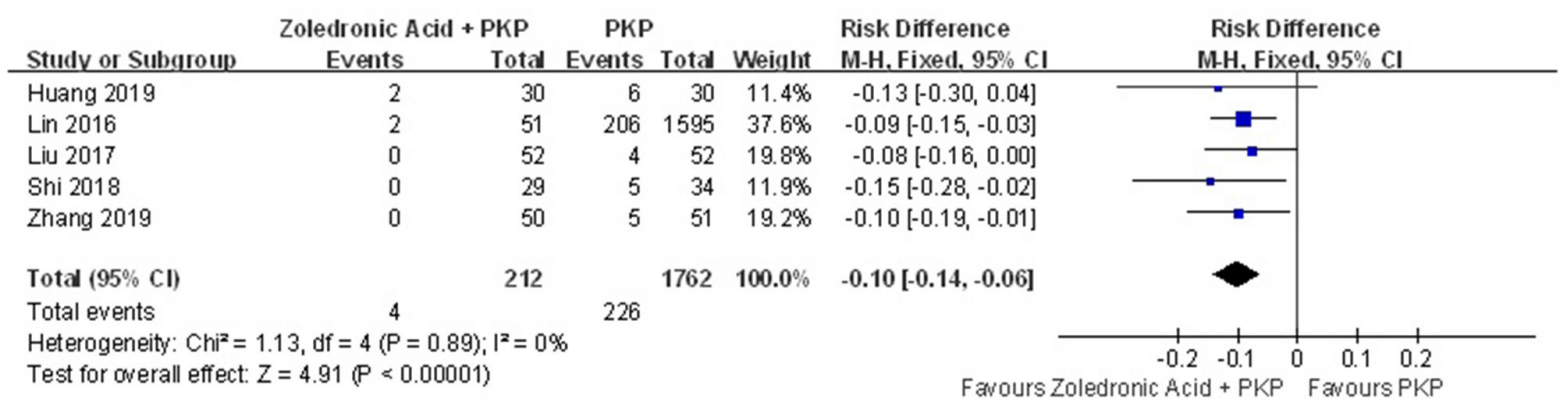
Forest plot showing additional vertebral body fractures.

Additional vertebral body fractures were assessed in five studies. The pooled results demonstrated that zoledronic acid combined with PKP was related to a significant decrease in the occurrence of additional vertebral body fractures compared with PKP alone (RD = −0.10; 95% CI, −0.14, −0.06; *P* < 0.00001), with no heterogeneity (n.s., *I*^2^ = 0%) (Figure 3).

#### Pre-operative and Post-operative Bone Mineral Density

Pre-operative and post-operative BMD were reported in four studies. For pre-operative BMD, there was no significant difference between the two groups (MD = 0.01; 95% CI, −0.02, 0.05; *P* = 0.44), with no heterogeneity (n.s., *I*^2^ = 27%) (Table 3). The pooled results demonstrated that compared with PKP alone, zoledronic acid combined with PKP significantly improved BMD at the 6- and 12-month follow-ups (Table 3).

**Table 3:** Meta-analysis results.

**Outcome**	**Studies**	**Groups (ZOL/PKP)**	**Overall effect**			**Heterogeneity**	
			**Effect estimate**	**95% CI**	***p*-value**	***I^**2**^*(%)**	***p*-value**
**BMD**							
Pre-operation	4	161/167	0.01	−0.02, 0.05	0.44	27	0.25
6 months	3	132/133	0.04	0.02, 0.07	0.0001	34	0.22
12 months	4	161/167	0.30	0.11, 0.48	0.001	95	0.00001
**VAS**							
Pre-operation	4	161/167	0.11	−0.04, 0.26	0.16	0	0.39
1 week	3	131/137	−0.12	−0.48, 0.24	0.52	67	0.05
6 months	3	109/115	−0.39	−0.77, −0.01	0.04	81	0.005
12 months	4	161/167	−0.80	−1.24, −0.35	0.0004	87	0.00001
**ODI**							
Pre-operation	2	81/86	−0.49	−1.77, 0.79	0.45	0	0.91
1 week	2	81/86	−1.32	−4.83, 2.19	0.46	89	0.002
12 months	2	81/86	−7.65	−8.88, −6.42	0.00001	49	0.16
**N-MID**							
Pre-operation	2	102/103	−0.59	−2.19, 1.01	0.47	23	0.25
6 months	2	102/103	−5.04	−7.71, −2.37	0.0002	66	0.09
12 months	2	102/103	−4.53	−5.68, −3.38	0.00001	53	0.14
**β-CTX**							
Pre-operation	2	102/103	−0.01	−0.06, 0.04	0.76	0	0.80
6 months	2	102/103	−0.12	−0.17, −0.07	0.001	49	0.06
12 months	2	102/103	−0.07	−0.09, −0.05	0.001	42	0.08

*BMD, Bone mineral density; VAS, Visual analog scale; ODI, Oswestry disability index; N-MID, N-terminal molecular fragment; β-CTX, beta collagen degradation product; ZOL, Zoledronic acid; PKP, Percutaneous kyphoplasty; CI, confidence interval*.

#### Pre-operative and Post-operative Visual Analog Scale

The pre-operative and post-operative VAS scores were documented in four studies. For the pre-operative and post-operative 1-week VAS scores, there were no significant differences between the two groups (Table 3). The pooled results demonstrated that compared with PKP alone, zoledronic acid combined with PKP significantly decreased the VAS score at the 6- and 12-month follow-ups (Table 3).

#### Pre-operative and Post-operative Oswestry Disability Index

The pre-operative and post-operative ODI scores were provided in two trials. For the pre-operative and post-operative 1-week ODI scores, there were no significant differences between the two groups (Table 3). The pooled results demonstrated that compared with PKP alone, zoledronic acid combined with PKP significantly improved the ODI score at the 12-month follow-up (Table 3).

#### Pre-operative and Post-operative N-Terminal Molecular Fragment

The pre-operative and post-operative N-MID values were available in two studies. For the pre-operative N-MID values, there was no significant difference between the two groups (Table 3). The pooled results demonstrated that compared with PKP alone, zoledronic acid combined with PKP significantly improved N-MID at the 6- and 12-month follow-ups (Table 3).

#### Pre-operative and Post-operative Beta Collagen Degradation Product (β-CTX)

Two articles reported the pre-operative and post-operative β-CTX values. For the pre-operative β-CTX values, there was no significant difference between the two groups (Table 3). The pooled results demonstrated that compared with PKP alone, zoledronic acid combined with PKP significantly improved β-CTX at the 6- and 12-month follow-ups (Table 3).

## Discussion

Our meta-analysis included five studies. The purpose of our meta-analysis was to evaluate the efficacy and safety of zoledronic acid combined with PKP in the treatment of OVCFs. In this analysis of studies, we found that compared with PKP alone, zoledronic acid combined with PKP decreased the incidence of additional vertebral body fractures, relieved low back pain, increased BMD and improved quality of life. To our knowledge, the present study is the first quantitative meta-analysis to evaluate the efficacy and safety of zoledronic acid combined with PKP in the treatment of OVCFs.

BPs have been confirmed to prevent bone loss and increase bone quality and density by inhibiting osteoclast-mediated bone resorption (4, 15). Zoledronic acid is administered by annual intravenous infusions (16). Therefore, zoledronic acid is the most commonly used and powerful BP in clinical practice (6). N-MID and β-CTX, which are bone metabolism markers, have been widely used in the diagnosis of osteoporosis, the monitoring of the effects of anti-bone resorption therapy and the prediction of osteoporotic fracture risk (17, 18). The pooled results suggest that zoledronic acid significantly decreases the post-operative levels of N-MID and β-CTX BMD at 6 and 12 months. Zoledronic acid can effectively decrease bone absorption.

During PKP, a balloon is percutaneously inflated in the fractured vertebral body to restore the height and kyphosis angle of the fractured vertebra. Then, bone cement is injected into the fractured vertebral body to maintain vertebral strength and stiffness (19). However, several studies have shown that vertebral augmentation (the injection of bone cement) leads to increased mechanical pressure on vertebrae adjacent to the region of PKP and may cause an additional vertebral body fracture (20–22). On the other hand, Rho et al. found that the major risk factors for new adjacent OVCFs include the progression of osteoporosis after the PKP procedure (23). The administration of BPs as anti-osteoporotic drugs has been confirmed to effectively prevent bone loss and increase bone quality and density. Several studies have verified that BPs decrease the risk of vertebral and non-vertebral fractures (5, 24). The present meta-analysis revealed that zoledronic acid not only significantly improved post-operative BMD at 6 and 12 months but also decreased the incidence of additional vertebral body fractures. The administration of zoledronic acid is beneficial for OVCFs treated with PKP.

Post-operative function and pain determine the overall efficacy of spinal surgery. The present meta-analysis suggests that zoledronic acid combined with PKP significantly improved the ODI score at the 12-month follow-up (MD = −7.56, *P* = 0.00001). The VAS score is a patient-reported score and has been used extensively in previous studies to assess patient pain following spinal surgery. The pooled data showed that BPs combined with PKP decreased the VAS score for back pain at the 6- and 12-month follow-ups. This small MD was thus quite unlikely to be the actual difference between the two groups. We should consider these factors when analyzing the present findings.

Several potential limitations should be noted. (1) Only three RCTs and 2 non-RCTs were included, and the sample size of all the studies was relatively small; (2) the suboptimal methodological quality of the included studies and insufficient outcomes may weaken our analysis; (3) we failed to perform subgroup analysis and determine the source of heterogeneity for the limited number of studies that were included.

## Conclusion

Among patients with OVCFs, zoledronic acid combined with PKP was associated with a higher BMD, a better quality of life, less severe low back pain, and fewer additional vertebral body fractures than was PKP alone.

## Data Availability Statement

The original contributions presented in the study are included in the article/Supplementary Material, further inquiries can be directed to the corresponding author/s.

## Author Contributions

All authors listed have made a substantial, direct and intellectual contribution to the work, and approved it for publication.

## Conflict of Interest

The authors declare that the research was conducted in the absence of any commercial or financial relationships that could be construed as a potential conflict of interest.
